# Hunger, Food Security, and Sovereignty: A Need for Evidence-Based Public Health Approaches to Meet Sustainable Development Goals

**DOI:** 10.3389/ijph.2023.1605956

**Published:** 2023-08-22

**Authors:** Olumide Arigbede, Oluwaseun Kilanko, Oluwatomilola Joy Arigbede, Olayemi Matthew

**Affiliations:** ^1^ Department of Epidemiology and Biostatistics, College of Pharmacy & Pharmaceutical Sciences, Institute of Public Health, Florida A&M University, Tallahassee, FL, United States; ^2^ Division of Economic, Social and Administrative Pharmacy, College of Pharmacy & Pharmaceutical Sciences, Institute of Public Health, Florida A&M University, Tallahassee, FL, United States; ^3^ Department of Nursing, Division of Healthcare Professions, Tallahassee Community College, Tallahassee, FL, United States

**Keywords:** hunger, food insecurity, evidence-based public health, sustainable development goals (SDGs), Sub-Saharan Africa (SSA)

Hunger remains one of the twenty-first century’s most critical public health concerns and only worsens. About 800 million people worldwide suffer from hunger and food insecurity, and 1 in 9 people sleep hungry because of food insecurity, inadequate finances to purchase food or a lack of equitable access to food [1]—more than 110 million people need food and nutrition [1]. In particular, hunger and food insecurity are associated with poor academic and behavioral performance in children and are responsible for more than 50% of all fatalities in children under five [2]. Without adequate public health methods, the Sustainable Development Goals (SDGs) to abolish global hunger by 2030 seem impossible. Theoretical concepts can slowly eradicate hunger, but evidence-based public health (EBPH) will prove efficient and guarantee food security.

**FIGURE 1:**
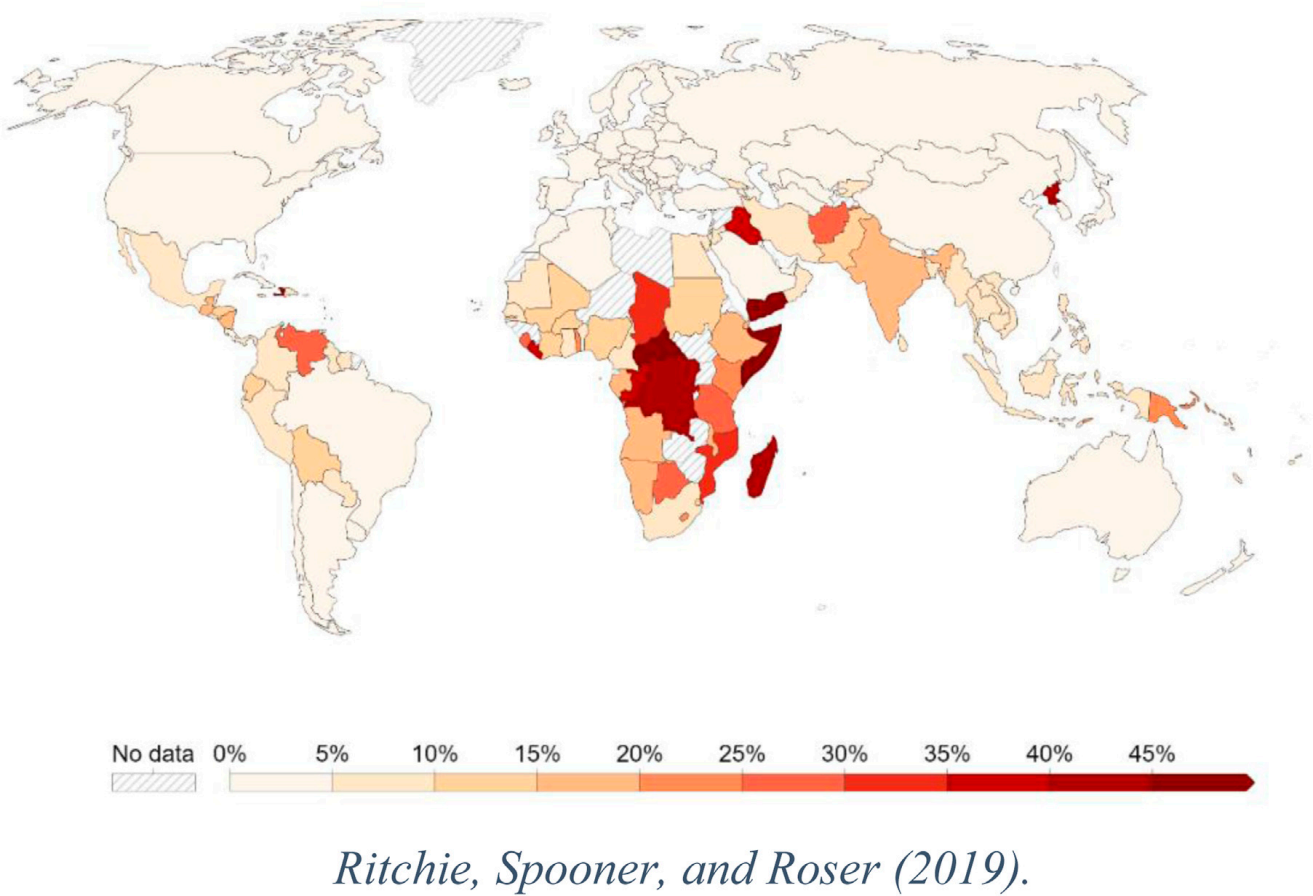
Global distribution of undernourished population (Oxford, England. 2019).

The continued hunger, food insecurity, and food sovereignty syndemic propose measures to mitigate their impacts, particularly among vulnerable people in low- and middle-income countries (LMICs). Food insecurity is difficulty obtaining sufficient, nutritious, and culturally appropriate food for active and healthy living [2]. Before the coronavirus disease 2019 (COVID-19) pandemic, global populations suffer from varying degrees of food insecurity and hunger, necessitating immediate intervention [2]. However, hunger and food shortages worsened in the preceding 3 years due to COVID-19 [2] and put global health at risk. Undoubtedly, hunger and food insecurity are global challenges that disproportionately affect young and low-income children, unemployed, and people with disabilities, among others, especially those living in LMICs in Sub-Saharan Africa (SSA) and South Asia regions (Figure 1) [2–3]. In addition, the consequences of hunger and food insecurity substantially impact the global health system, increasing the likelihood of poor health outcomes such as cancer, obesity, anxiety, depression, memory loss, and other infectious and chronic diseases [3]. For instance, obesity, a global problem that doubled in prevalence rates between 1975 and 2016, and causes about 2.8 million deaths yearly, is linked to food insecurity [3]. The global distribution of hunger and food insecurity-related mortality is disheartening, signaling the need for immediate public health intervention.

EBPH combines public health initiatives and science-based approaches with an informed, precise, and conscientious use of evidence to improve population health using evidence-based decisions [4]. Because EBPH provides access to excellent information on proven methods that work and an increased likelihood of improved outcomes if the appropriate policies are enacted, the current global frameworks for food security and nutrition to combat hunger and ensure food availability, access, utilization, and stability will function well using EBPH [4].

EBPH strategies can be beneficial to abolishing hunger as discussed hereto: 1) Investing in education and food security programs is crucial to combat food insecurity and hunger. When developing the SDGs, the United Nations recognized the importance of education in achieving all goals, including eliminating hunger and food insecurity [5]. Research by the UTSA Urban Education Institute further highlights the strong association between education and food insecurity [6]. By investing in food security initiatives, vulnerable populations can be empowered; as a result, food access and availability will improve. Food security initiatives such as participating in during-school, after-school, and summer meal programs that provide meals at no cost or a discount to qualifying pupils, as well as creating school food pantries programs that supply fresh food to students and their families [5–6], can improve access to education, eliminate hunger, and enhance academic engagement, particularly among school-aged children [5]. In addition, adequate dietary education, such as accurately labeling food packages in grams and calories, can aid in the alleviation of food insecurity [7] and hence, improve healthy food choices; 2) Developing a reliable and accurate food surveillance system is a critical tool to alleviate food insecurity and hunger by providing real-time information on food production, availability, distribution, and consumption. This information can detect regions where food is limited, keep track of food costs, predict hunger surges among vulnerable populations, and more effectively prioritize nutritional initiatives. In the United States (US), for example, hospitals and health systems can screen patients for food insecurity and collaborate with a coalition of community groups to offer initiatives and amenities that promote access to nutritious foods and create community awareness [8]. A food surveillance system can also promote research that fosters food security through policy review. Finally, a reliable surveillance system can help in the efficient administration of food and nutrition assistance programs, such as the Supplemental Nutrition Assistance Program (SNAP), targeted at eliminating food insecurity [2] and preventing foodborne disease outbreaks so that people have access to nutritious and healthy food; 3) Strengthening of sociopolitical systems can be achieved through improving food system management, making inclusive and accountability-driven investments in rural communities, empowering small-scale farmers, and strengthening social protection measures for risk management. Some examples include boosting agricultural productivity, supplementing foods to provide undernourished populations with specific micronutrients, and protecting the human rights of populations susceptible to hunger and malnutrition [6]. Effective governments should serve as the pillars of good governance that promote equitable food systems for communities, reducing food security disparities; 4) Improving fresh produce availability and accessibility can eradicate food insecurity by boosting the availability, accessibility, and affordability of healthful dietary alternatives. In many situations, food insecurity—lack of access to excess healthy food for active well-being [2] is closely linked to poverty and constrained access to healthy foods [6]. People can access healthier food alternatives by boosting the availability and accessibility of fresh produce, which can help them maintain a balanced diet and lower the risk of chronic conditions, malnutrition, and other health problems [9]. Strategies to increase the availability and accessibility of fresh produce include community farms, farmers’ markets, roadside shopping trolleys, and federal programs like SNAP and Women, Infants, and Children (WIC) nutrition programs. These initiatives promote healthy diets, create green spaces, and provide fresh produce to those without access to grocery stores or supermarkets [2, 10]; and 5) Tackling structural problems through evidence-based government intervention and transparency will end food insecurity and hunger by encouraging justice and providing integrity through equitable access to food [10]. According to Miller and Thomas (2020), behind forward-thinking governance principles, a view of structural factors as fundamental causes of food insecurity and hunger and the notion that access to food is an ethical issue requiring government attention are critical to reducing food insecurity [10]. The US can completely restructure all affiliated institutions that manage hunger and food insecurity by employing a cross-sectoral strategy to eliminate hunger. However, evidence-based structural measures can be difficult to translate into improved health or reduced health disparities because improving the importance, utilization, and delivery of evidence-based strategies in real-world settings requires time, resources, and collaboration among experts [4]. The Evidence-Based Policymaking Act of 2018 (Evidence Act) recognizes that federal decision-makers require evidence, including performance data, program assessment, and research, to determine whether government programs achieve the desired outcomes [11].

Understanding the systemic and fundamental factors contributing to food insecurity and malnutrition is critical for identifying and prioritizing activities to enhance food security and nutrition. Furthermore, as a public health issue, the solution to accomplishing the second goal of the 17 2030 SDGs, to eradicate hunger [9], rests in appropriating EBPH approaches. In conclusion, EBPH interventions can fully address and provide proven solutions to eradicate hunger and food insecurity.
